# Carboxymethyl Chitosan and Gelatin Hydrogel Scaffolds Incorporated with Conductive PEDOT Nanoparticles for Improved Neural Stem Cell Proliferation and Neuronal Differentiation

**DOI:** 10.3390/molecules27238326

**Published:** 2022-11-29

**Authors:** Shui Guan, Yangbin Wang, Feng Xie, Shuping Wang, Weiping Xu, Jianqiang Xu, Changkai Sun

**Affiliations:** 1 State Key Laboratory of Fine Chemicals, School of Chemical Engineering, Dalian University of Technology, Dalian 116024, China; 2 Dalian R&D Center for Stem Cell and Tissue Engineering, Dalian University of Technology, Dalian 116024, China; 3 Research & Educational Center for the Control Engineering of Translational Precision Medicine (R-ECCE-TPM), School of Biomedical Engineering, Faculty of Electronic Information and Electrical Engineering, Dalian University of Technology, Dalian 116024, China; 4 School of Ocean Science and Technology & Panjin Institute of Industrial Technology, Dalian University of Technology, Panjin 124221, China; 5 School of Life and Pharmaceutical Sciences, Dalian University of Technology, Panjin 124221, China

**Keywords:** conductive hydrogel, poly(3,4-ethylenedioxythiophene), carboxymethyl chitosan, gelatin, neural stem cells, in situ interfacial polymerization

## Abstract

Tissue engineering scaffolds provide biological and physiochemical cures to guide tissue recovery, and electrical signals through the electroactive materials possess tremendous potential to modulate the cell fate. In this study, a novel electroactive hydrogel scaffold was fabricated by assembling poly(3,4-ethylenedioxythiophene) (PEDOT) nanoparticles on a carboxymethyl chitosan/gelatin (CMCS/Gel) composite hydrogel surface via in situ chemical polymerization. The chemical structure, morphology, conductivity, porosity, swelling rate, in vitro biodegradation, and mechanical properties of the prepared hydrogel samples were characterized. The adhesion, proliferation, and differentiation of neural stem cells (NSCs) on conductive hydrogels were investigated. The CMCS/Gel-PEDOT hydrogels exhibited high porosity, excellent water absorption, improved thermal stability, and adequate biodegradability. Importantly, the mechanical properties of the prepared hydrogels were similar to those of brain tissue, with electrical conductivity up to (1.52 ± 0.15) × 10^−3^ S/cm. Compared to the CMCS/Gel hydrogel, the incorporation of PEDOT nanoparticles significantly improved the adhesion of NSCs, and supported long-term cell growth and proliferation in a three-dimensional (3D) microenvironment. In addition, under the differentiation condition, the conductive hydrogel also significantly enhanced neuronal differentiation with the up-regulation of β-tubulin III expression. These results suggest that CMCS/Gel-PEDOT hydrogels may be an attractive conductive substrate for further studies on neural tissue repair and regeneration.

## 1. Introduction

Tissue engineering is aiming to develop functional constructs that combine biomaterial scaffolds, precursor cells, and growth factors to restore and improve damaged tissues. A major challenge of this approach is obtaining a stable source of healthy cells that develop into mature functional tissues [1,2]. In this regard, stem cells have been proven as an alternative source of personalized regenerative medicine. As a pluripotent cell population, neural stem cells (NSCs) are exhibiting promise for various brain cancers, traumas, and even neurodegenerative disorders [3]. The NSC line can self-renew and differentiate into neurons and other glial cells (astrocytes and oligodendrocytes), which are capable of integrating with host tissues and repairing nerve damage through the improvement of neurogenesis and axon growth [4]. Especially, external biophysical stimulations have shown promising effects in modulating cellular functionalities through biomaterial scaffolds. This is of great importance for the design of “smart” biomaterials able to act as multifunctional platforms, delivering both cells and therapeutic drugs to the damaged region while providing biological and physiochemical cures to support tissue recovery [5,6,7]. A series of studies have shown that the proliferation and differentiation of NSCs can be regulated using multiple factors, such as elastic stiffness, surface topography, and mechanical and electrical signals at the cell–matrix interface [8,9,10]. However, the implementation of these approaches remains a challenge due to our limited comprehension of the phenomena occurring in both healthy and damaged host tissues.

Electrical signals are well known to possess tremendous potential to regulate cell fate and cellular processes [1,11]. Electrogenic cells such as neural and cardiac cells can make use of endogenous electric fields to transmit electrical signals/impulses [12,13]. It has been demonstrated that the use of electrical stimulation (ES) to control redox events at the cellular interface will regulate intercellular signaling, leading to customized cellular functionalities [14]. Electrical currents with various intensities and patterns can lead to different biological effects, such as enhancing the proliferation, migration, and differentiation of stem cells into neural cells, and ES-based therapies may regenerate functional native tissue faster [15,16]. This also makes exogenous electrical stimulation a viable treatment option for neurological disorders (e.g., deep brain therapy in Parkinson’s disease) [17]. Along with advances in medicine and molecular biology, the functional properties of the engineered biomaterial scaffolds as the primary factor to determine cellular behavior have received great attention [18].

Recent studies advocated combined strategies that coordinate well-defined interactions between physical and biochemical cures to influence the cell fate decisions [19,20]. As a particular type of scaffold, conductive polymer hydrogel scaffolds are currently interesting for their additional advantage of promoting cell growth by electrical stimulation [1,21,22]. Various conductive polymers, such as polyalinine (PANI), polypyrrole (PPy), and poly(3,4-ethylenedioxythiophene) (PEDOT), have been used to provide a synthetic conducting environment for cell proliferation and neurite outgrowth [23]. Numerous studies have shown that the electroactivity of biomaterials influences the protein adsorptions and regulates cell proliferation and differentiation. It is well established that they support modulated tissue regeneration by delivering localized bioelectrical cues at the implantation site [24,25]. Conductive polymer scaffolds can be synthesized by many techniques and, in particular, PEDOT scaffolds have been prepared by chemical, vapor-phase, and electrochemical polymerization of 3,4-ethylenedioxythiophene (EDOT) monomer around a previously synthesized scaffold [26,27,28,29]. Therefore, by incorporating conducting polymer nanoparticles into the natural or synthetic biomaterials, the electrical conduction of scaffolds may increase, while maintaining biocompatibility and supporting precursor cell growth and differentiation.

Chitosan (Cs) is a linear polysaccharide with randomly distributed β-(1–4)-linked D-glucosamine (deacetylated unit) and N-acetyl-D-glucosamine (acetylated unit), and features good moisturizing and adsorption properties [30]. Gelatin (Gel) is derived from naturally occurring collagen by hydrolysis and can be used as a cheaper replacement while maintaining its good biodegradability, compatibility with living tissue, cell adhesion, and proliferation properties [31]. Both polymers have strong hydrogen bonds inside and between them, which contribute to the formation of a blend with enhanced mechanical properties compared to individual ones [32]. Particularly, due to the possible formation of durable hydrogen bonds during the preparation of samples, the combination of Cs with Gel led to higher tensile strength and improved the biodegradation kinetics of the Cs/Gel scaffolds [33]. In addition, Cs blends with the addition of Gel also cause much greater cell adhesion than each of the materials separately [34]. In our previous studies, a porous Cs/Gel scaffold containing hyaluronic acid (HA) and heparan sulfate was fabricated via a freeze-drying technique, which was able to assist the adhesion of NSCs and their differentiation into neuronal and glial cells well [35]. Through assembling conductive PEDOT components into the Cs/Gel macromolecular network, we further developed the novel PEDOT/Cs/Gel [26] and PEDOT-HA/Cs/Gel [36] composite scaffolds. The incorporation of PEDOT nanoparticles significantly increased the electrical conductivity and mechanical properties, indicating that PEDOT-assembled electroactive scaffolds may have more potential for neural tissue engineering applications. However, it is worthwhile to mention that Cs is insoluble in water and most organic solvents, seriously restricting both application scope and applicable fields. Therefore, the modification of Cs has been regarded as an important aspect of its research, such as for better solubility, pH-sensitive targeting, and increased numbers of delivery systems [30]. 

Carboxymethyl chitosan (CMCS) is an amphoteric and water-soluble Cs derivative with many excellent properties, including water solubility, water retention, biodegradability, biocompatibility, and antibacterial and antioxidant activity that contribute to wider applications than for Cs in the medical healthcare and biochemical fields [30,37]. The carboxylation reaction mainly utilizes glyoxylic acid or chloroalkanoic acid to react with the C_6_-OH or C_2_-NH_2_ groups of Cs, obtaining N-CMCS, O-CMCS, or N-O-CMCS as products, which greatly improves its solubility, biodegradation, and biocompatibility [38]. Interestingly, CMCS can be easily modified due to the functional -OH, -NH_2_, and -COOH groups, and thus has also received extensive attention in neural tissue engineering applications [27,39]. For instance, it has been demonstrated that 1-ethyl-3(3-dimethylaminopropyl) carbodiimide hydrochloride (EDC) covalently cross-linked CMCS films or tubes exhibited good biocompatibility with Neuro-2a and Schwann cells, and provided better nerve regeneration performance than that of Cs for repairing sciatic nerve injury in rats [40,41]. Moreover, a novel composite chitin/CMCS artificial nerve graft was constructed and applied to bridge sciatic nerve regeneration across a 10 mm defect with a similar effect compared to the autograft in SD rats [42]. Therefore, it can be speculated that CMCS amalgamated with Gel could be more suitable for neural tissue engineering applications than Cs/Gel scaffold, and even more applicable for synthetic conductive scaffolds.

In the current study, we carried out the fabrication of a novel conductive hydrogel by incorporating conductive PEDOT nanoparticles into the CMCS/Gel matrix. The precross-linked CMCS/Gel hydrogel was used as a template and PEDOT was uniformly assembled on the CMCS/Gel porous scaffold channel surface via in situ interfacial polymerization. The chemical structure, morphology, electrical conductivity, porosity, swelling rate, in vitro biodegradation, and mechanical properties were investigated. Furthermore, NSCs were seeded to evaluate the biocompatibility of the conductive hydrogel scaffolds. The results showed that CMCS/Gel-PEDOT hydrogels were conducive to cell adhesion, proliferation, and neuronal differentiation of NSCs, indicating that the hydrogels may be applicable as an attractive conductive substrate for neural tissue repair and regeneration.

## 2. Results and Discussion

### 2.1. Synthesis of CMCS/Gel-PEDOT Conductive Hydrogels

**Figure 1:**
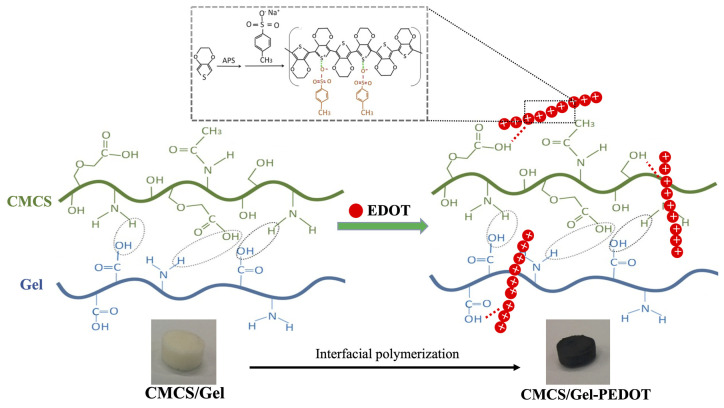
Schematic diagram of the synthesis of CMCS/Gel-PEDOT hydrogels.

CMCS/Gel-PEDOT hydrogels were fabricated in this study by assembling PEDOT nanoparticles on porous CMCS/Gel scaffold surface via an in situ interfacial polymerization method. The schematic diagram of the synthesis of CMCS/Gel-PEDOT hydrogels is shown in Figure 1. After washing gently several times with 70% alcohol and deionized water, the CMCS/Gel-PEDOT hydrogel scaffolds were lyophilized and sterilized for the following experiments. Scaffolds were prepared with three different molar concentration ratios of APS/pTS-Na to EDOT (1:1, 1:1.5, 1:2), and the control samples underwent the same procedure but without EDOT monomer. Four types of hydrogel scaffolds were obtained (Table 1), namely CMCS/Gel-0EDOT or CMCS/Gel (control), CMCS/Gel-0.1EDOT, CMCS/Gel-0.15EDOT, and CMCS/Gel-0.2EDOT, respectively.

### 2.2. FTIR Spectra Analysis of Hydrogels and PEDOT Nanoparticles

**Figure 2:**
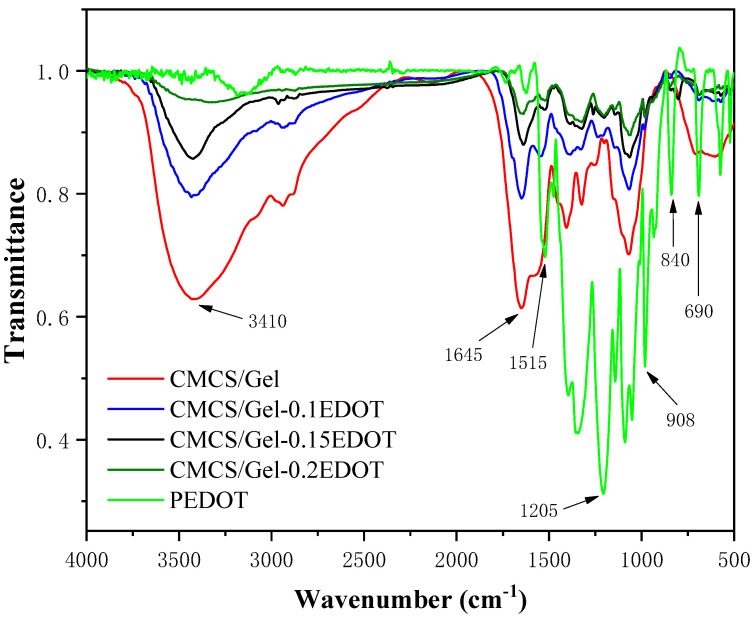
FTIR spectrogram of PEDOT nanoparticles, CMCS/Gel and CMCS/Gel-PEDOT hydrogels.

The FTIR spectra of CMCS/Gel, CMCS/Gel-0.1EDOT, CMCS/Gel-0.15EDOT, CMCS/Gel-0.2EDOT, and PEDOT are shown as curves in Figure 2, respectively. The N-H stretching vibration peak of the amide bond appeared near 3410 cm^−1^, and the C=O stretching vibration peak of the amide bond appeared at 1645 cm^−1^, indicating that CMCS and Gel cross-linked to form an amide bond. Compared to the FTIR of CMCS/Gel, hydrogels doped with different contents of PEDOT all have the characteristic peaks of PEDOT: the C-S-C bond stretching vibration peak of the thiophene ring appears at 690 cm^−1^, 840 cm^−1^, and 981 cm^−1^; the asymmetric stretching vibration peak of the C=C bond of the thiophene ring appears at 1515 cm^−1^; 1205 cm^−1^ is the bending vibration peak of the ethylenedioxy ring C-O-C bonds on the thiophene ring [27,43]. These results indicate that the EDOT monomer was successfully oxidatively polymerized in situ on the CMCS/Gel hydrogel.

### 2.3. Morphology of Hydrogels

**Figure 3:**
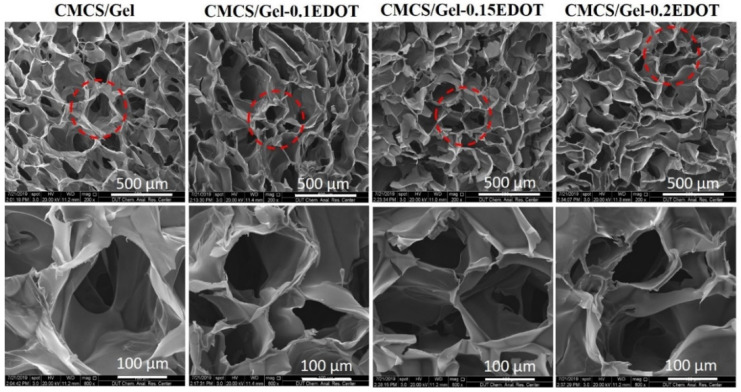
SEM images of CMCS/Gel and CMCS/Gel-PEDOT hydrogels.

The microstructure of hydrogels is crucial for the life metabolism of cells. The open porous and interconnected network is beneficial to promote cell feeding, adhesion, proliferation, migration, etc., which is conducive to the vascularization and formation of new tissue [44]. It is well known that the source of materials, cross-linking reagent concentration, manufacturing procedure, and posttreatment directly influence the morphological properties of hydrogels [45]. The SEM images of the hydrogels are shown in Figure 3. All samples have a highly porous structure with circular and elliptical pores uniformly distributed throughout the structure. The average pore size of the samples was about 50–250 μm, which facilitates the input of nutrients and the output of metabolic waste from cells, as well as the exchange of information between cells. Similar to CMCS/Gel scaffolds, there was no significant difference in the microscopic morphologies of the three conductive scaffolds, indicating that EDOT is uniformly polymerized on the surface of the pore walls of the CMCS/Gel hydrogel, and has no significant effect on the pore structure and pore size of the scaffolds.

### 2.4. Electrical Conductivity of Hydrogels

Currently, studies on enhancing the electrical activity of tissue engineering scaffolds by incorporating conductive polymers have received extensive attention. Numerous reports have demonstrated that conductive hydrogels can improve signaling and chemical exchange between cells, which is beneficial to cell growth and tissue regeneration [46]. In this work, we investigated the electrical conductivities of the prepared conductive hydrogels using a standard four-probe technology in the swollen state, which reflects the actual conductivity of the hydrogel when used as a scaffold for tissue engineering. The results are listed in Table 1. It was shown that, due to ionic conductivity of carboxyl groups and amine groups on the main chain of CMCS/Gel, the conductivity of CMCS/Gel hydrogel is about (3.14 ± 0.36) × 10^−6^ S·cm^−1^. With the increase in EDOT content in the CMCS/Gel hydrogels, the conductivity of CMCS/Gel-PEDOT hydrogels increased linearly from (8.67 ± 0.27) × 10^−5^ S·cm^−1^ to (1.52 ± 0.15) × 10^−3^ S·cm^−1^, indicating that high PEDOT content generally makes it easier to form a conductive network that benefits the electron transfer. As known, polythiophene polymers are electroactive due to the conjugation of π-electrons formed by the overlapping of carbon p-orbital along the backbone [47]. Usually, the oxidation polymerization of EDOT proceeds with a reaction stoichiometry of 2.33 mol of electrons per mole of monomer, and the monomer/oxidant molar ratio has a major role in the electroconductivity properties of the conductive polymers [48]. In this experiment, the PEDOT-assembled CMCS/Gel hydrogels exhibited better electrical conductivity when the molar ratio of EDOT monomer to APS/pTS-Na was increased, which is basically consistent with our previous studies [26,27]. However, it is worth pointing out that the continuous increase in the ratio of EDOT to APS/pTS-Na may also lead to the accumulation of excess electrons, thereby increasing the oxidation potential in the reaction system, resulting in a decrease in conductivity (data not shown).

### 2.5. Porosity and Swelling Rate of Hydrogels

**Figure 4:**
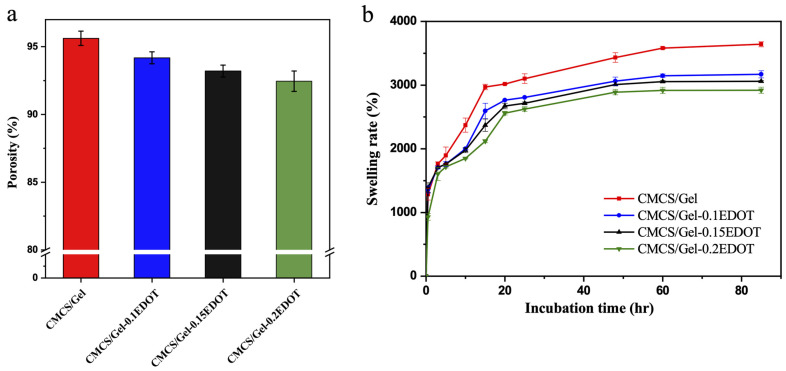
The porosity (**a**) and swelling rate (**b**) of CMCS/Gel and CMCS/Gel-PEDOT hydrogels.

The high porosity of the hydrogels provides sufficient stereoscopic space for cell growth and enables the transport of nutrients and removal of wastes [46]. In this study, the porosity of hydrogel samples was calculated and results are shown in Figure 4a. The CMCS/Gel hydrogel had a higher porosity of (95.62 ± 0.53)%, and the porosity of conductive scaffolds as a whole decreased with the increase in the mass concentration of PEDOT. As can be seen, the porosities of CMCS/Gel-0.1EDOT, CMCS/Gel-0.15EDOT and CMCS/Gel-0.2EDOT hydrogels were (94.18 ± 0.44)%, (93.2 ± 0.44)%, (92.52 ± 1.62)%, respectively. However, there was no significant difference in porosity compared with the CMCS/Gel group. The results might be because the stiffness of the PEDOT polymer tightened the porous network, resulting in a more compact structure and ultimately a decrease in porosity [27]. In general, all prepared samples contained very high porosity and the incorporation of the PEDOT component into CMCS/Gel hydrogels had no appreciable effect on the porosity.

Likewise, the swelling properties of the hydrogels are important for their biomedical applications, and the swelling rate is the criterion for describing water absorption of the hydrogels [49]. The swelling rate of CMCS/Gel-PEDOT hydrogels is shown in Figure 4b. Due to the repulsion between the -COO- groups from the CMCS and Gel matrix, the CMCS/Gel hydrogel showed the highest swelling rate, which increased dramatically to (2900 ± 460)% in the first 24 h, and then slowly up to (3500 ± 240)% in the next 48 h. The CMCS/Gel-PEDOT hydrogels showed a similar swelling kinetic compared to CMCS/Gel hydrogel. However, the water absorption ability of conductive hydrogels decreased accordingly with increasing the amount of PEDOT in the hydrogels. It is well known that the water absorption capacity of a hydrogel is related to the hydrophilicity of the material. Due to the hydrophobic nature of conductive polymer, incorporation of that into the hydrophilic hydrogel tends to reduce the hydrophilicity of the network. On the other hand, the hydrophilic groups of CMCS/Gel (-COOH, -NH_2_, and -OH) were partially covered by PEDOT nanoparticles on the hydrogel surface, which may also lead to a decrease in hydrophilicity [26]. In the current study, the swelling rate of the CMCS/Gel-PEDOT hydrogels is much higher than that of PEDOT/CMCS hydrogels in the previous work [27], implying that these hydrogels may have a broader potential for tissue engineering applications, since the water absorption of hydrogels is beneficial for the transport of cells, nutrition, oxygen, and wastes, during tissue repair and regeneration.

### 2.6. Thermal Stability and Biodegradation of Hydrogels

**Figure 5:**
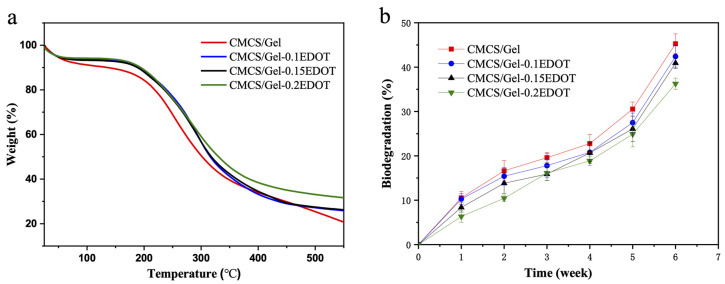
TGA curves (**a**) and biodegradation (**b**) of CMCS/Gel and CMCS/Gel-PEDOT hydrogels.

The thermal stability of hydrogels was determined by TGA and the results are shown in Figure 5a. When the temperature increased, the weight of hydrogels decreased gradually, and the main weight loss occurred from 150–450 °C. Interestingly, the residual weight of CMCS/Gel-PEDOT hydrogels was more than that of CMCS/Gel hydrogel at the same temperature, especially the scaffolds with higher PEDOT content. For example, when the temperature exceeded 300 °C, the residual weight of the CMCS/Gel-0.2EDOT hydrogel was significantly higher than that of other samples, indicating that the incorporation of PEDOT nanoparticles can enhance the thermodynamic properties of the CMCS/Gel hydrogel.

Meanwhile, the prepared hydrogel for tissue engineering should have a suitable biodegradation rate that is coordinated with the healing rate of the host tissue, since ideal scaffolds are typically required to be degraded or reabsorbed by the body after successful tissue regeneration [49]. In this study, the CMCS/Gel-PEDOT hydrogels were incubated in an enzymatic solution containing lysozyme at 37 °C for 6 weeks to evaluate the weight loss in the scaffolds due to enzymatic degradation. As illustrated in Figure 5b, the conductive hydrogels were biodegradable and the degradation gradually increased over the incubation time. It was very straightforward to observe that the weight loss of the CMCS/Gel hydrogel was relatively faster than that of the CMCS/Gel-PEDOT groups that had slower degradation rate, notably for those with higher amounts of PEDOT. The CMCS/Gel hydrogel lost approximately (45.27 ± 2.21)% in weight at week 6, while the CMCS/Gel-0.2EDOT hydrogel showed about (36.26 ± 1.27)% weight loss, indicating that the presence of PEDOT significantly improved the stability and the biodegradation resistance of the CMCS/Gel hydrogels. The results can probably be attributed to the incorporation of PEDOT nanoparticles on the surface of the CMCS/Gel hydrogel, resulting in a decrease in the contact area of aqueous enzyme in the hydrogel network, thereby reducing the amount of enzyme entering the pores of the hydrogel. On the other hand, the partial loss of hydrophilicity in the conductive hydrogels leads to the decrease in water absorption, which reduces the permeability of the water containing enzyme into the hydrogels. This can also explain why the biodegradation of the CMCS/Gel-PEDOT hydrogels is slower than that of the CMCS/Gel hydrogel.

### 2.7. Mechanical Properties of Hydrogels

The mechanical properties of the native extracellular matrix (ECM) play a key role in regulating cell behavior during development, healing, and homeostasis. With respect to the biomaterial mechanical signals, it has been demonstrated that manipulating them can promote changes in cell behaviors such as spreading, proliferation, migration, and differentiation [50,51]. Therefore, mimicking the brain ECM microenvironment by engineering hydrogels capable of coping with some mechanical stress from adjacent tissues and maintaining at least a slight elasticity and bendability without collapse or losing their shape may allow for investigation and better understanding of NSC behavior.

**Figure 6:**
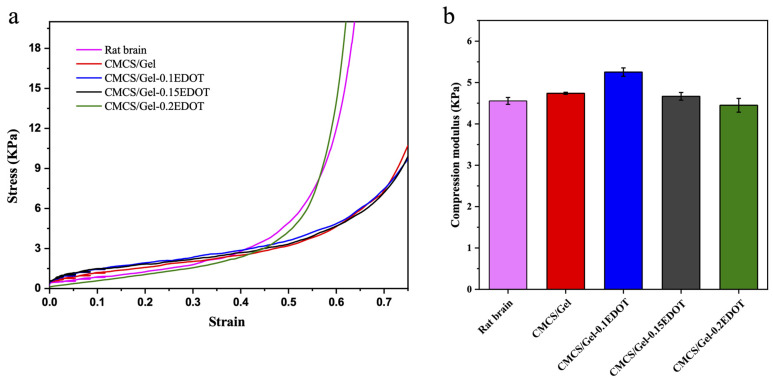
Stress–strain curves (**a**) and compression modulus (**b**) of CMCS/Gel, CMCS/Gel-PEDOT hydrogels and the brain tissue of newborn SD rat.

In the present study, the CMCS/Gel and the CMCS/Gel-PEDOT hydrogels in the hydrated state exhibited elasticity similar to that of pure rat brain tissue. All the hydrogels showed excellent compressive properties, and the extruded hydrogels could return to their original shape immediately after reabsorbing water. It was shown that the stress–strain curves (Figure 6a, up to 70% strain) and the compressive modulus (Figure 6b), taken from the 0~0.38 strain region of the CMCS/Gel-PEDOT conductive hydrogels, were remarkably consistent with those of the brain tissue, showing typical viscoelasticity curve characteristics. Compared to the group without PEDOT, the conductive hydrogels did not show a significant difference in compressive modulus with an increase in PEDOT content, indicating that incorporation of PEDOT does not destroy the integrity of the microstructure of the CMCS/Gel matrix. Overall, the prepared hydrogels have mechanical properties suitable for actual brain tissue and are expected to be used as ideal materials for neural stem cell culture and transplantation.

### 2.8. The Adhesion and Viability of NSCs in the CMCS/Gel-PEDOT Hydrogels

**Figure 7:**
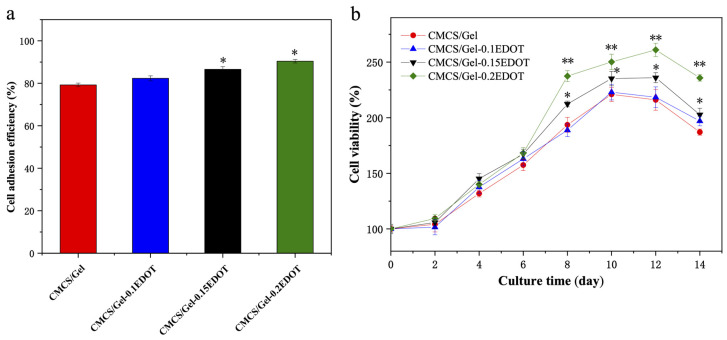
Cell adhesion efficiency (**a**) and proliferation (**b**) of NSCs in CMCS/Gel and CMCS/Gel-PEDOT hydrogels. Data are mean ± SD values obtained from four culture wells per experiment, determined in three independent experiments, and (*) *p* < 0.05, (**) *p* < 0.01, statistically different from the CMCS/Gel group.

The cell adhesive response to the biomaterial surface is one of the most important properties of biocompatible materials, and the evaluation of cell adhesion in scaffolds can reveal the contribution of various components of the hydrogels in cell surface contacts. As demonstrated previously, the CMCS/Gel-PEDOT conductive hydrogels exhibited excellent water absorption, high porosity, and large surface area similar to the extracellular matrix environment, which are favorable for cell adhesion and proliferation. In this experiment, NSCs were first incubated in the conductive hydrogels for a period of 4 h to evaluate the extent of initial cell attachment. As illustrated in Figure 7a, compared to the CMCS/Gel scaffolds, the number of cells attached to the CMCS/Gel-PEDOT hydrogel scaffolds was significantly increased. With the increase in PEDOT content, the adhesion efficiency of NSCs in the conductive hydrogels increased, and the CMCS/Gel-0.15EDOT and CMCS/Gel-0.2EDOT groups were significantly different from the CMCS/Gel group (* *p* < 0.05). The results showed that the addition of PEDOT into the CMCS/Gel hydrogels not only increased the roughness of the structure surface, but also positively affected cell adhesion, promoting overall cell retention, especially at the initial point of culture. This phenomenon could probably be explained as that the introduction of PEDOT weakens the strong water absorption of the hydrogel, making the inner walls of the hydrogel pores more favorable for cell adhesion. It is well known that the extracellular side of the cell membrane carries a net negative charge due to the dense and confluent negatively charged network of proteoglycans, glycolipids, and glycoproteins that contribute to intercellular recognition, communication, and adhesion. Conductive polymers, as stimulated responsive materials, can partially dissolve along the electrochemical potential, thereby releasing cations into solution, which may affect ion flux, sealing resistance, and thus extracellular membrane potential fluctuations [52,53]. These findings indicated that after the PEDOT nanoparticles were incorporated in the CMCS/Gel matrix, the favorable electrical activity of CMCS/Gel-PEDOT hydrogels also had a positive effect on cell adhesion.

Furthermore, the ideal biomaterial scaffolds should be biocompatible, safe to use, and have very low or no cytotoxicity, to support long-term 3D culture in vitro. In order to determine whether PEDOT-assembled CMCS/Gel hydrogels are beneficial for NSC growth and proliferation, the cell viability of NSCs on the CMCS/Gel-PEDOT hydrogels was evaluated by the CCK-8 assay over a long period of time. The results are shown in Figure 7b. With the prolongation of culture time, the proliferation of NSCs in CMCS/Gel-PEDOT hydrogel scaffolds tended to be higher than those in the CMCS/Gel hydrogel. In particular, after being cultured for 12 days, the CMCS/Gel-0.15EDOT and the CMCS/Gel-0.2EDOT scaffolds caused the highest ((236.41 ± 4.51)% and (261.02 ± 5.97)%, respectively) increase in cell viability, although the cell viability of all samples decreased to some extent after 14 days. These results demonstrated that the conductive hydrogels have better cytocompatibility compared with CMCS/Gel hydrogel, which are more favorable for cell growth and proliferation.

### 2.9. The Differentiation of NSCs in the CMCS/Gel-PEDOT Hydrogels

**Figure 8:**
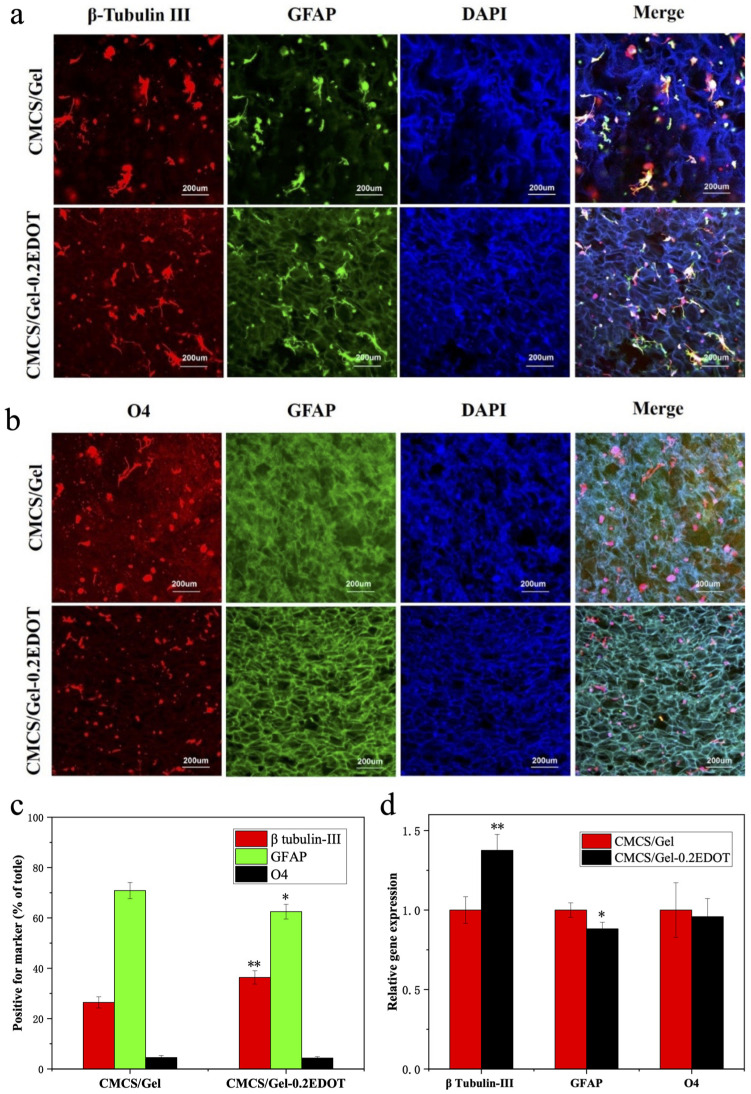
The differentiation of NSCs in CMCS/Gel and CMCS/Gel-PEDOT hydrogels after culture in the proliferation medium for 5 days and in the differentiation medium for 3 days. Neurons are immunostained with β-tubulin III (red), astrocytes are immunostained with GFAP (green), and oligodendrocytes are immunostained with O4 (red). (**a**) The fluorescence immunostaining images for β-tubulin III and GFAP; (**b**) the fluorescence immunostaining images for O4 and GFAP; (**c**) quantification of cells positive for the markers; (**d**) RT-qPCR analysis of the gene expression levels for neurons, astrocytes, and oligodendrocytes. Data are mean ± SD values, and (*) *p* < 0.05, (**) *p* < 0.01, statistically different from the CMCS/Gel group.

Previous work has established that the NSCs used in this study differentiate into three types of neural cells, neurons, astrocytes, and oligodendrocytes, in the differentiation medium of 1% FBS without EGF and bFGF [54,55]. To determine whether NSCs cultured in the CMCS/Gel-PEDOT hydrogels may retain their hallmark ability to generate mature differentiated cells, after being cultured in the proliferation medium for 5 days, NSCs were grown in the differentiation condition for an additional 3 days and stained for lineage-specific markers by immunofluorescence. Neurons were immunostained with β-tubulin III (red), astrocytes were immunostained with GFAP (green), and oligodendrocytes were immunostained with O4 (red). As shown in Figure 8a,b, β-tubulin III-, GFAP-, and O4-positive cells were observed on the CMCS/Gel and CMCS/Gel-0.2EDOT hydrogels, indicating that NSCs maintained the pluripotency to differentiate into three types of neural cells. Compared with the CMCS/Gel group, the percentage of neurons (β-tubulin III-positive) in the CMCS/Gel-0.2EDOT group was significantly increased, and the percentage of astrocytes (GFAP-positive) was significantly decreased. However, the oligodendrocytic (O4-positive) percentage was found to show no significant difference between the different hydrogels as shown in Figure 8c. Meanwhile, we also tested the differentiation of NSCs in the CMCS/Gel and CMCS/Gel-0.2EDOT hydrogels using the normal proliferation medium, however, hydrogels alone were not sufficient to guide the differentiation of NSCs (data not shown).

In addition, the quantitative gene expression was assayed to further confirm this result. After being cultured for 3 days in the differentiation medium, NSCs were collected from CMCS/Gel and CMCS/Gel-0.2EDOT hydrogels to analyze the neural gene expression levels by using the RT-qPCR method. As shown in Figure 8d, the relative gene expression of β-tubulin III in cells in the CMCS/Gel-0.2EDOT hydrogel was (1.38 ± 0.10)-fold, higher than that in the CMCS/Gel hydrogel. Consistent with the quantitative analysis of marker-positive cells, the GFAP expression was slightly lower (0.88 ± 0.04), while the expression of the O4 gene did not show significant difference between the different hydrogels. These results further confirmed that the incorporation of conductive PEDOT nanoparticles into the CMCS/Gel hydrogel plays a crucial role for the differentiation of NSCs into neurons.

NSCs have great potential to serve as a cell reservoir to restore damaged neural tissues, recover impaired cells, and regain lost biological function. However, direct transplantation of NSCs is a huge challenge and, since abnormal cell architectures develop in vivo, cells have limited survival upon implantation, and it is difficult for them to directly differentiate into specific phenotypes [56,57]. Conductive hydrogels can address these gaps through their softness and malleability by providing the necessary structural support and physicochemical cues to facilitate neural cell proliferation, attachment, differentiation, neurite extension, and migration [58,59]. The interaction between the hydrogel components and materials is crucial, as enhanced entrapment can ensure the molecules do not escape unless an electric field affects the polymer matrix structure. Hence, it is important to optimize the interactions between the conductive materials and polymers, and also optimize the payloads to tune properties toward desired electroresponsive behavior and cellular interactions [60,61,62]. In our future study, the effect of conductive CMCS/Gel-PEDOT hydrogels on NSCs combined with electrical stimulation will be investigated for further regulating the directional differentiation of NSCs.

## 3. Materials and Methods

### 3.1. Materials

Chitosan (Cs, 85.6% deacetylation, 50 mPa.s) was obtained from Haidebei Marine Bioengineering Co. Ltd. (Jinan, China) and gelatin (Gel, with 99.5% purity) was purchased from Solarbio Co. Ltd. (Beijing, China). 3,4-ethylenedioxythiophene (EDOT, with 99.9% purity) monomer was supplied by Shanghai Macklin Chemical Co. Ltd. (Shanghai, China). The cross-linkers, 1-ethyl-3-(3-dimethylaminopropyl) carbodiimide hydrochloride (EDC), N-hydroxy succinimide (NHS), and 2-morpholinoethanesulfonic acid (MES), were purchased from Shanghai Medpep Co. Ltd. (Shanghai, China). All other reagents and solvents were of analytical grade. Cell culture reagents were obtained from Gibco Life Technologies (Carlsbad, CA, USA). The Cell Counting Kit-8 (CCK-8) was purchased from Dojindo Laboratories (Kumamoto, Japan) and Live-Dead Cell Staining Kit was supplied by Sigma-Aldrich Inc. (St. Louis, MO, USA). 4’,6-diamidino-2-phenylindole (DAPI) and antibodies for NSCs were purchased from Abcam Inc. (Cambridge, MA, USA).

### 3.2. Preparation of CMCS

Carboxymethyl chitosan (CMCS) was prepared according to a modified procedure described previously [27]. In brief, CS (8 g) and NaOH (10.8 g) were added into a mixture of water (16 mL) and isopropanol (64 mL) to swell and alkalize for 3 h at 35 °C. Next, chloroacetic acid (12 g) was dissolved in isopropanol (16 mL), and added into the previous mixture dropwise for 30 min. After continued stirring for an additional 3 h at 35 °C, ethanol (200 mL) was added to stop the reaction, and the filtered solid product (sodium salt of CMCS) was thoroughly rinsed with 80% ethanol several times to desalinize and dehydrate until the filtrate pH was neutral, and then vacuum-dried overnight at 100 °C. The degree of substitution (DS) of the carboxymethyl group was measured by potentiometric titration [63]. By recording the volume of NaOH consumed at the pH jump point, the DS of CMCS was calculated to be 79%, and the average molar mass of CMCS basic units was estimated to be 230. The obtained white product was used to prepare CMCS/Gel and subsequent CMCS/Gel-PEDOT hydrogel in the next experiments.

### 3.3. Preparation of CMCS/Gel-PEDOT Conductive Hydrogels

CMCS/Gel-PEDOT hydrogels were produced by assembling PEDOT nanoparticles on porous CMCS/Gel scaffold surface via an in situ interfacial polymerization method as our laboratory previously described [26,27]. Briefly, CMCS solution (2%, *w*/*v*) and Gel solution (2%, *w*/*v*) were firstly mixed with a ratio of 2:1 (*v*/*v*). The mixture was purified by 1500 r/min centrifugation for 5 min to remove trace insoluble impurities and bubbles, pipetted into 24-well plates (about 1.5 mL/well), and then frozen at −80 °C for 24 h. The frozen samples were further lyophilized for 48 h in a freeze-dryer (FreeZone18, Labconco, USA) to produce the porous hydrogels. Then, the hydrogels were cross-linked in an ethanol/MES buffer (80%, *v*/*v*, pH 6.0) containing 50 mM of EDC and NHS. After incubation at room temperature (RT) for 24 h, the hydrogels were rinsed overnight with plenty of deionized water to remove the residual cross-linking agent, and finally relyophilized for 24 h to obtain the porous CMCS/Gel hydrogel scaffolds. Next, the precross-linked CMCS/Gel hydrogel scaffolds were immersed in an aqueous solution containing ammonium persulfate (APS, 0.1 M, as an oxidant) and sodium p-toluenesulfonate (pTS-Na, 0.1 M, as a dopant) for 3 h under vacuum, to assure the uniform dispersion of reagent in hydrogel scaffolds. EDOT precursor solution was prepared by mixing different concentrations of EDOT monomer in n-hexane (0.1, 0.15, and 0.2 M), and the CMCS/Gel samples (7 mm × 7 mm × 2 mm) were then transferred into EDOT/hexane solution to accomplish the in situ polymerization of EDOT. The polymerization was carried out in a vibrator with a slight vibration at RT for 72 h, to accelerate the diffusion of EDOT monomer from organic phase to aqueous phase, and also to effectively lengthen the PEDOT chain for the formation of PEDOT-pTS second network on the surface of hydrogel scaffolds. With the white CMCS/Gel hydrogel scaffold becoming black, PEDOT nanoparticles were assembled in situ on the CMCS/Gel scaffold channel surface.

### 3.4. Fourier Transform Infrared Spectroscopy

Fourier transform infrared (FTIR) spectra of CMCS/Gel hydrogel, conductive CMCS/Gel-PEDOT hydrogels, and PEDOT nanoparticles were measured and recorded on an FTIR spectrometer (Nicolet 6700, Thermo Fisher, Waltham, MA, USA) at room temperature. Briefly, CMCS/Gel hydrogel, CMCS/Gel-0.1EDOT, CMCS/Gel-0.15EDOT, CMCS/Gel-0.2EDOT, and PEDOT nanoparticles were put in 4 mL tubes and then frozen down by placing into liquid nitrogen for 5 min. Then, samples were taken out from the liquid nitrogen and immediately mashed into powder with a glass rod. Finally, the samples were vacuum-dried for 12 h to obtain completely dry powder samples. For each FTIR spectrum analysis, the hydrogel samples were separately mixed with KBr and then tested at wavenumber from 4000 cm^−1^ to 500 cm^−1^ with a spectral resolution of 4 cm^−1^.

### 3.5. Scanning Electron Microscope

The morphology of the hydrogel scaffolds was characterized by environmental scanning electron microscopy (ESEM, QUANTA 450, FEI, Hillsboro, OR, USA). The samples were sliced, mounted on an aluminum SEM stub with double-sided carbon tape, sputter coated with gold for 90 s, and images were taken at 3 kV. The pore size of scaffolds was analyzed from the SEM images and the diameters of pores in each of 10 random fields per specimen were measured.

### 3.6. Electrical Conductivity

A four-probe electrical conductivity meter (Keithley 2400 Standard Series SMU, Tektronix, Beaverton, OR, USA) was used to determine the conductivity of the CMCS/Gel-PEDOT hydrogel scaffolds. For each sample, three specimens’ conductivity was measured 6 times and averaged.

### 3.7. Porosity

The porosity of the hydrogel scaffolds was calculated by an ethanol infiltration method. Briefly, the weight of dry scaffolds and a pycnometer filled with anhydrous ethanol were recorded as *m*_d_ and *m*_1_, respectively. Then, the dry scaffolds were immersed into the pycnometer, and maintained for 15 min under reduced pressure, to force the ethanol to completely penetrate into the hydrogel pores. After the solution was refilled, the total weight of the pycnometer was recorded as *m*_2_. Then, the scaffolds were taken out and the weight including the pycnometer and remaining solution was recorded as *m*_3_. Each scaffold was analyzed in triplicate and the porosity of the scaffolds was calculated according to the following Equation (1):

(1) $$Porisity\;(\%)=\frac{m2-m3-md}{m1-m3}\times 100\%$$

### 3.8. Swelling Rate

The swelling rate of the hydrogel scaffolds was also determined as described in our previous work [27]. The weight of dry scaffold was taken as *m*_0_. After immersion into PBS solution to absorb water completely, the wet weight of scaffold was recorded as *m*_s_. All tests were performed in triplicate and the swelling rate of the scaffolds was calculated using the following Formula (2):

(2) $$Swelling\;rate\;\left(\%\right)=\frac{ms-mo}{mo}\times 100\%$$

### 3.9. Thermogravimetric Analysis (TGA)

A thermogravimetric analyzer (TG, X70, Netzsch, Selb, Germany) was used for the thermal stability analysis of the hydrogel scaffolds. Sample masses were around 5–10 mg and the experiments were carried out at a heating rate of 10 °C/min under nitrogen atmosphere at a temperature range from 25 to 550 °C. A ceramic crucible was used in this experiment. 

### 3.10. In Vitro Enzymatic Biodegradation

For the degradation assay, the hydrogel scaffolds were impregnated with a sterile PBS buffer (pH 7.45) containing 2 mg/mL lysozyme and placed in a 37 °C, 5% CO_2_ incubator for 6 weeks. The medium was replaced every week. The detailed steps were performed according to our previous work [27].

### 3.11. Compressive Modulus

In this experiment, we investigated the mechanical properties of the different PEDOT contents of hydrogel scaffolds, with the brain tissue of newborn SD rats (18 h) as a control. All animals used in this study were used in accordance with protocols and guidelines approved in “Instructive notions with respect to caring for laboratory animals” for animal experimentation by the Ministry of Science and Technology of the People’s Republic of China in 2006. All efforts were made to minimize the suffering and number of animals. The SD rats were decapitated, the brain was removed from the skull and stored in ice-cold carbogen saturated artificial cerebrospinal fluid. Slices were cut in a horizontal plane with a thickness of approximately 2 mm using a VT1200S vibratome (Leica Biosystems, Nussloch, Germany). The compressive modulus of each sample was assessed by using an electromechanical universal testing machine (SANS CMT4204, MTS, Guangzhou, China). The compressive testing was performed in unconfined conditions and a compression force was loaded at a displacement control loading rate of 1 mm/min. The size of scaffold (dry and hydrated) was 7 mm × 7 mm × 2 mm and all tests were performed in triplicate. The specimens of CMCS/Gel and CMCS/Gel-PEDOT hydrogel scaffolds were hydrated prior to mechanical testing in PBS at 37 °C for 48 h, and then loaded under a compression clamp and tested for controlled strain percentage (ramping from 0 to 80%) with preloaded force of 0.01 N and displacement of 10 μm. The slope of the stress–strain curve from 20 to 40% strain was used to determine compressive modulus. The compressive modulus was calculated as the tangent slope of the stress (*σ*) and the strain (*ε*) according to the following Equation (3):

(3) $$Compression\;modulus\;\left(\mathrm{KPa}\right)=\frac{\sigma }{\varepsilon }=\frac{Load\;Value/A}{Position\;Value/h}\times 1000\left(\frac{\mathrm{MPa}}{\mathrm{KPa}}\right)$$

### 3.12. NSC Culture

The NSCs were isolated and purified from the hippocampus of fetal Sprague Dawley rats (gestation days 13–15, provided by Experimental Animal Center of Dalian Medical University in China). Briefly, the cells with suspended neurospheres were cultured in proliferation medium containing DMEM/F12 with 20 ng/mL bFGF (Peprotech, Cranbury, NJ, USA), 20 ng/mL EGF (Peprotech, Cranbury, NJ, USA), and 2% B27 supplement (Gibco, Gaithersburg, MD, USA) and maintained in a humidified atmosphere with 5% CO_2_ at 37 °C. When the diameter of the neurospheres reached about 100–150 μm, neurospheres were collected by enzymatically digesting with Accustase^TM^ (Sigma-Aldrich, St. Louis, MO, USA) to obtain single cell suspension for passages. The primary cells were referred to as passage 0 and NSCs at a passage from 4 to 10 were used for all experiments. For NSC differentiation, cells were cultured in differentiation medium containing DMEM/F12 with 1% N2 supplement (Gibco, Gaithersburg, MD, USA), 1 μM retinoic acid (RA, Sigma-Aldrich, St. Louis, MO, USA), and 1% fetal bovine serum (FBS, Gibco, Carlsbad, CA, USA) instead of bFGF and EGF.

### 3.13. Cell Viability Assay

To evaluate the biocompatibility of hydrogels, NSCs were seeded into the CMCS/Gel and CMCS/Gel-PEDOT hydrogel scaffolds for 3D culture in vitro with the proliferation medium or differentiation medium, respectively. Before cell seeding, the scaffolds were fixed on the bottom of a 24-well or 96-well plate and rinsed with 75% ethanol for 20 min. After washing three times with PBS, the single cell suspension of NSCs (20 μL) was injected into the hydrogel scaffolds at a density of 1 × 10^6^ cells/mL and incubated in a 5% CO_2_ humidified incubator at 37 °C for 4 h. The cell adhesion efficiency on CMCS/Gel and CMCS/Gel-PEDOT hydrogel scaffolds was calculated as the cell number percentage of cells attached onto the scaffold to the total number of cells initially injected into the scaffold (4 × 10^4^ cells per scaffold). At a certain time point, cell viability was evaluated by using CCK-8 assay (Dojindo, Kumamoto, Japan) according to the manufacturer’s instructions. Absorbance was measured at 450 nm by a multifunctional microplate reader (SpectraMax M2e, Molecular Devices, Silicon Valley, CA, USA).

### 3.14. Immunocytochemistry Analysis

After culturing for a defined time under differentiation conditions, the cells in scaffolds were fixed with ice-cold 4% paraformaldehyde for 20 min and washed thrice with PBS for 10 min. After blocking with 1% bovine serum albumin (BSA, Sigma-Aldrich, St. Louis, MO, USA) in PBS containing 0.1% Triton X-100 (Sigma-Aldrich, St. Louis, MO, USA) for 1 h, the specimens were incubated with primary antibodies at 4 °C overnight, followed by secondary antibodies for 1 h. The primary antibodies included mouse antirat β-tubulin III (1:1000, Abcam, Cambridge, MA, USA), rabbit antirat GFAP (1:1000, Abcam, Cambridge, MA, USA), and mouse antirat O4 (1:100, Abcam, Cambridge, MA, USA). The secondary antibodies included goat antirabbit Alexa Fluor 488 (1:1000, Abcam, Cambridge, MA, USA) and goat anti-mouse Alexa Fluor 647 (1:1000, Abcam, Cambridge, MA, USA). Next, the nuclei were stained with DAPI (Abcam, Cambridge, MA, USA) for 15 min. Finally, the specimens were washed with PBS three times and imaged with laser scanning confocal microscopy (LSCM, FV1000, Olympus, Tokyo, Japan). The number of immunostained cells was counted and the ratio of positive cells was calculated in each of 10 random fields per specimen.

### 3.15. Gene Expression Analysis

Gene expression related to differentiation of NSCs was also analyzed by quantitative real-time polymerase chain reaction (RT-qPCR) as described in our previous work [54]. Briefly, each hydrogel scaffold seeded with NSCs was cultured in the proliferation medium for 5 days, followed by an additional 3 days in differentiation medium. RNA was extracted from cells in the hydrogel scaffolds using an RNA extraction kit (Takara Bio Inc., Otsu, Shiga, Japan). Next, RNA was reverse transcribed into cDNA using a PrimeScript™-RT Master Mix reverse transcription kit (Takara Bio Inc., Otsu, Shiga, Japan) according to the manufacturer’s instructions. The qPCR assay was performed with SYBR Premix Ex Taq™ (Takara Bio Inc., Otsu, Shiga, Japan). The relative transcript expression levels of the target gene were normalized to GAPDH (internal control) and the results were analyzed using Rotor Gene Software 6.0.14.

### 3.16. Statistical Analysis

All quantitative data were presented as the mean ± standard deviation (SD) from three independent experiments and evaluated using one-way analysis of variance (ANOVA) followed by Student’s *t*-test. Differences at *p* < 0.05 were considered statistically significant.

## 4. Conclusions

In summary, combining the advantages of both PEDOT nanoparticles and CMCS/Gel hydrogel, a conductive CMCS/Gel-PEDOT hydrogel was fabricated via in situ chemical polymerization. The results demonstrated that the CMCS/Gel-PEDOT hydrogel had high porosity, excellent water absorption, improved thermal stability, and adequate biodegradability. Remarkably, the mechanical properties of the prepared hydrogels were similar to those of brain tissue, with electrical conductivity up to (1.52 ± 0.15) × 10^−3^ S/cm. Compared to the CMCS/Gel hydrogel, the incorporation of PEDOT nanoparticles significantly improved the adhesion of NSCs, and supported long-term growth and proliferation in the 3D microenvironment. Moreover, the CMCS/Gel-PEDOT hydrogel also significantly enhanced neuronal differentiation with the up-regulation of β-tubulin III expression under the differentiation condition. We demonstrate a novel electroactive CMCS/Gel-PEDOT hydrogel as an attractive conductive substrate for potential applications on neural tissue repair and regeneration.

## Figures and Tables

**Table 1:** Chemical oxidative polymerization and electrical conductivity of CMCS/Gel-PEDOT hydrogels with various ratios of EDOT monomer to APS/pTS-Na.

Hydrogel	Composition (M)			Reaction System (mL)	Conductivity (S·cm^−1^)
	pTS-Na	APS	EDOT		
CMCS/Gel	0.1	0.1	0	2	(3.14 ± 0.36) × 10^−6^
CMCS/Gel-0.1EDOT	0.1	0.1	0.1	2	(8.67 ± 0.27) × 10^−5^
CMCS/Gel-0.15EDOT	0.1	0.1	0.15	2	(5.54 ± 0.73) × 10^−4^
CMCS/Gel-0.2EDOT	0.1	0.1	0.2	2	(1.52 ± 0.15) × 10^−3^

## Data Availability

Not applicable.

## Sample Availability

Samples of the compounds are available from the authors.
